# Steakhouse syndrome

**DOI:** 10.1002/ccr3.4329

**Published:** 2021-06-09

**Authors:** Kiyoshi Shikino, Masatomi Ikusaka

**Affiliations:** ^1^ Department of General Medicine Chiba University Hospital Japan

**Keywords:** dysphagia, esophageal food impaction, steak

## Abstract

In Steakhouse syndrome, computed tomography revealed circumferential esophageal wall thickening and a mass in the esophageal lumen, which could be mistaken as esophageal cancer.

## Graphical abstract

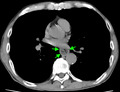

A 72‐year‐old man suddenly developed chest pain, dysphagia, and repetitive vomiting during dinner 4 days before presenting to our hospital. He had hypertension and type 2 diabetes mellitus but no history of specific diseases, including neurodegenerative diseases. We performed chest computed tomography to investigate the cause of chest pain, and it revealed circumferential esophageal wall thickening (Figure 1). Upper gastric endoscopy showed an insufficiently masticated piece of steak occluding the esophagus (Figure 2), which was removed using net forceps (Figure 3). No mechanical obstructive lesions or functional abnormalities were found in his esophagus, and his symptoms resolved without relapse.

**FIGURE 1:**
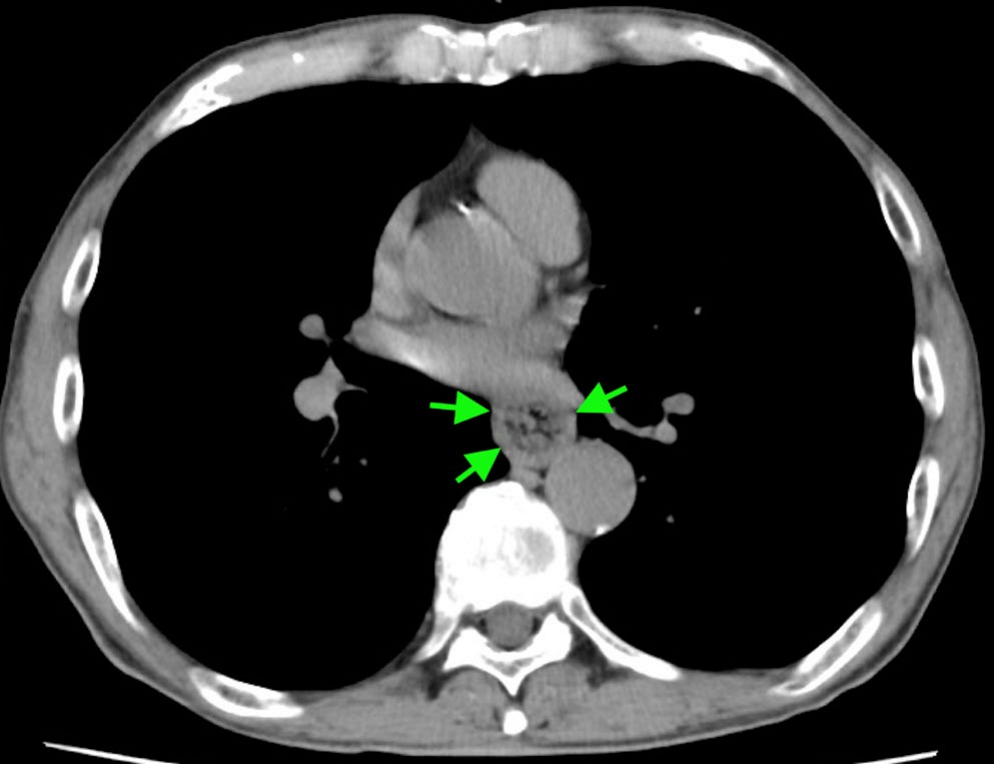
Computed tomography revealed circumferential esophageal wall thickening (arrows)

**FIGURE 2:**
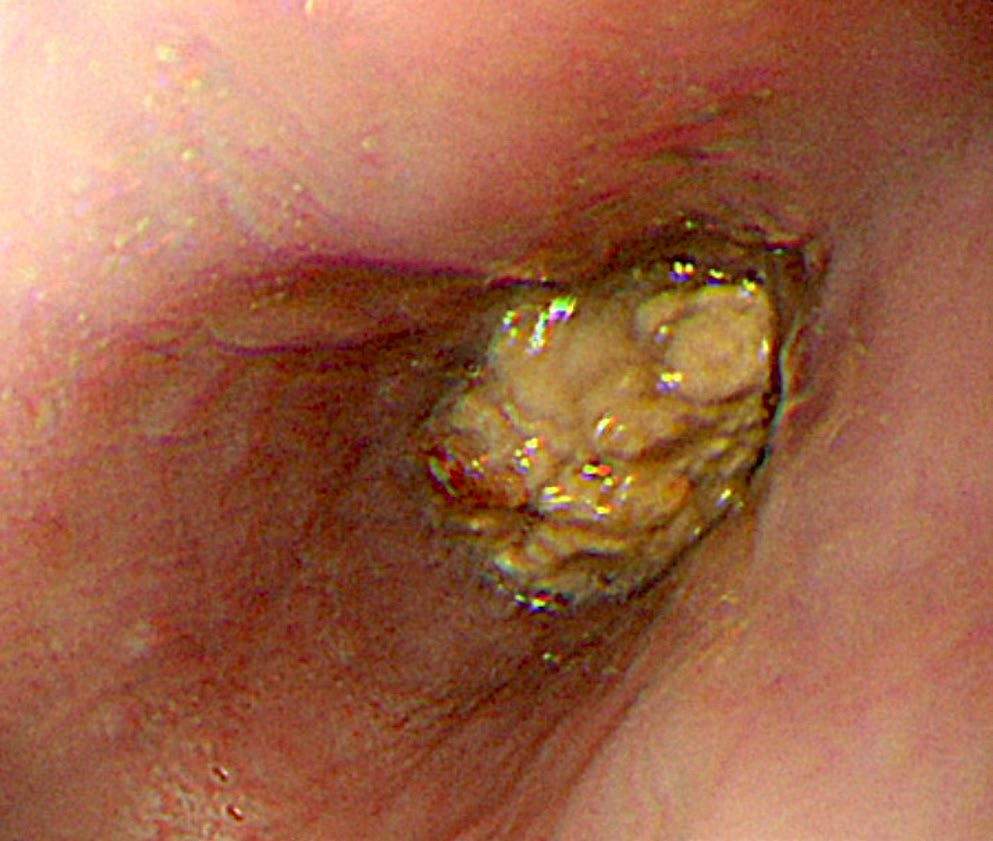
Upper gastric endoscopy showed an insufficiently masticated piece of steak occluding the esophagus

**FIGURE 3:**
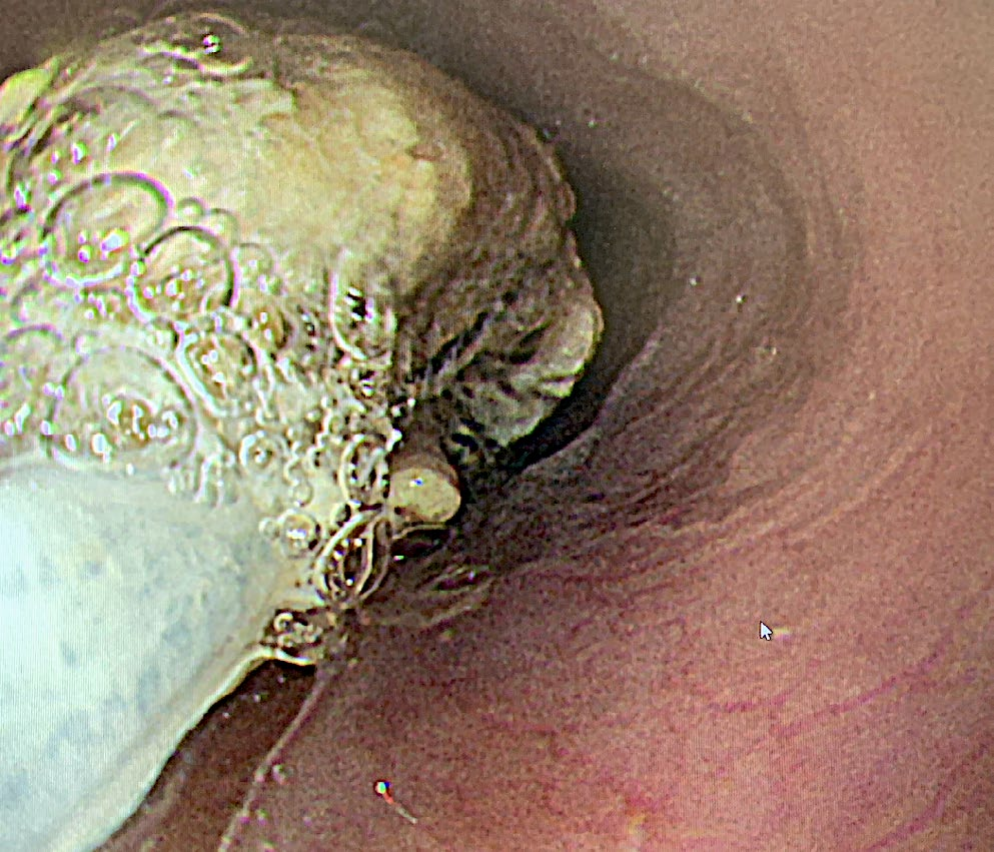
The removal of the piece of meat

Esophageal food impaction, known as “steakhouse syndrome,” is a condition in which food is consumed too fast and becomes stuck in the esophagus.^1^ It is more common in older patients.^1^ This syndrome can be caused by various etiologies: esophageal mechanical narrowing due to esophageal carcinoma, diverticulum, hiatal hernia, or eosinophilic esophagitis; or esophageal motility disturbances including esophageal achalasia, diffuse esophageal spasm, and esophagogastric junction outflow obstruction.^1^, ^2^, ^3^ This disease can be confused with acute coronary syndrome because the patient may complain of pain behind the sternum.^1^, ^4^ In this case, computed tomography revealed circumferential esophageal wall thickening and a mass in the esophageal lumen, which could be mistaken as esophageal cancer. A characteristic history is the most important clue to the diagnosis of steakhouse syndrome. For avoid food impaction, it is important to eat slowly and chew all food thoroughly before swallowing or take in a smaller amount of food per bite.

## CONFLICT OF INTEREST

None declared.

## AUTHOR CONTRIBUTION

KS: cared for the patient and wrote the first draft. KS and MI: read and approved the final version of the report. All authors had access to the data and a role in writing the manuscript.

## INFORMED CONSENT

We have obtained the consent of the patient for publication.

## Data Availability

Data sharing not applicable to this article as no datasets were generated or analyzed during the current study.
